# Discovery and Early Clinical Development of Selective Immunoproteasome Inhibitors

**DOI:** 10.3390/cells11010009

**Published:** 2021-12-21

**Authors:** Christopher J. Kirk, Tony Muchamuel, Jinhai Wang, R. Andrea Fan

**Affiliations:** Kezar Life Sciences, Inc., South San Francisco, CA 94080, USA; tmuchamuel@kezarbio.com (T.M.); jwang@kezarbio.com (J.W.); afan@kezarbio.com (R.A.F.)

**Keywords:** immunoproteasome, autoimmunity, KZR-616, immunomodulatory

## Abstract

Inhibitors of the proteolytic activity of the 20S proteasome have transformed the treatment of multiple B-cell malignancies. These agents have also been employed with success in the treatment of patients with autoimmune diseases and immune-mediated disorders. However, new agents are needed to fully unlock the potential of proteasome inhibitors as immunomodulatory drugs. The discovery that selective inhibitors of the immunoproteasome possess broad anti-inflammatory activity in preclinical models has led to the progression of multiple compounds to clinical trials. This review focuses on the anti-inflammatory potential of immunoproteasome inhibition and the early development of KZR-616, the first selective inhibitor of the immunoproteasome to reach clinical testing.

## 1. Introduction

**Figure 1:**
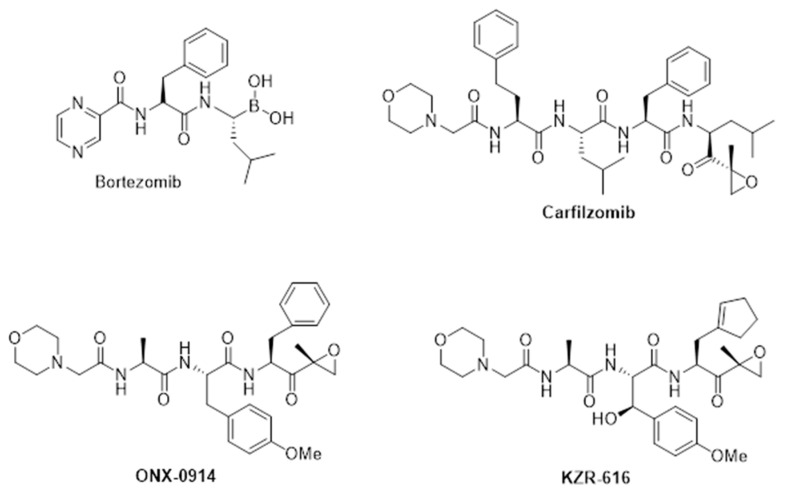
Structures of FDA-approved proteasome inhibitors (bortezomib and carfilzomib) and selective inhibitors of the immunoproteasome (ONX 0914 and KZR-616).

In 2004, Ciechanover, Hershko, and Rose were awarded the Nobel Prize in recognition of their seminal research that helped uncover and elucidate one of the most basic mechanisms of cellular function, the regulated turnover of intracellular proteins via the ubiquitin–proteasome system (UPS) [1]. It was no coincidence that this award came shortly after the first regulatory approval of a UPS-targeted agent, the boronic acid bortezomib (VELCADE™), for the treatment of relapsed and refractory Multiple Myeloma [2] (Figure 1). Through specific targeting of the active sites of the 20S proteasome, and thus the regulation of intracellular protein turnover, bortezomib revolutionized the treatment of this highly aggressive and lethal B-cell neoplasm, and its use has expanded to other B-cell tumors such as Waldenström Macroglobulinemia and Mantle Cell Lymphoma [3]. Subsequently, two other proteasome inhibitors, the peptide epoxyketone carfilzomib (KYPROLIS™) and the boronic acid ixazomib (NINLARO™) have been added to the cancer treatment armamentarium, and we have also learned that other classes of anti-cancer therapeutics such as immunomodulatory drugs (IMiDs–e.g., thalidomide, lenalidomide, and pomalidomide) exert their therapeutic effect via targeting of the UPS [4]. As is the case for many therapeutic classes, the road towards the application of proteasome inhibitors as anti-cancer agents was a winding one and continues to evolve in terms of our understanding of the molecular mechanisms and breadth of clinical application. Prior to clinical development in oncology, bortezomib was positioned as an anti-inflammatory agent and has more recently been used therapeutically in patients with refractory B-cell-driven immune-mediated disorders [5]. An extensive body of research suggests that bortezomib exerts an anti-inflammatory response via the inhibition of the immunoproteasome. In this review, we will trace the lineage from early research with the first-generation proteasome inhibitors to the discovery and early clinical development of selective inhibitors of the immunoproteasome designed with a specific purpose for use in immune-mediated disorders.

## 2. Anti-Inflammatory Activity of First-Generation Proteasome Inhibitors

The notion that the proteasome regulates immune responses became evident soon after the discovery of the first inhibitors of catalytic activity [6]. Using agents such as the β-lactone-based natural product, lactacystin, multiple roles of the proteasome in complex immune environments were quickly uncovered including T-cell activation, cytokine production by innate effector cells, and regulation of antigen presentation. Lactacystin was known to inhibit active site subunits in both the constitutive proteasome (β1, β2, and β5) as well as subunits of then newly described immunoproteasome (LMP2, MECL1, and LMP7). As research revealed that the proteasome was responsible for the activation of NF-κB [7], which initiated the transcription of multiple genes encoding inflammatory cytokines, and key components of the cell cycle [8], interest grew that proteasome inhibitors could induce an anti-inflammatory response in vivo. Scientists at Proscript (later Millennium Pharmaceuticals) developed an improved version of lactacystin, PS-519, as well as peptide-based proteasome inhibitors including the dipeptide boronic acid PS-341 (later bortezomib). These compounds, by dint of improved pharmaceutic properties, enabled testing in vivo and were profiled in several animal models of autoimmunity such as psoriasis, rheumatoid arthritis (RA), multiple sclerosis (MS), ischemia-induced inflammation, and transplant tolerance [6]. The breadth of therapeutic activity across these models stirred initial excitement that this ubiquitously expressed target could be harnessed to reduce inflammation in chronic conditions. However, toxicities in animals, presumably due to widespread tissue distribution and target inhibition, resulted in an unacceptable safety profile for development as a chronic treatment in non-life-threatening disorders [2]. The toxicity profile of bortezomib, coupled with compelling anti-tumor activity in mouse models steered developers to bring bortezomib forward as an anti-neoplastic agent [9].

Within five years of regulatory approval of bortezomib for the treatment of multiple myeloma, the first case studies of clinical application of proteasome inhibition in immune-mediated disorders began to emerge. Everly et al. applied short courses of bortezomib treatment to patients undergoing antibody-mediated rejection (AMR) and acute cellular rejection (ACR) of their kidney transplants [10]. In all six patients, donor-specific antibody (DSA) levels decreased rapidly after administration of bortezomib, reminiscent of reductions of tumor-specific immunoglobulin (M-protein) in myeloma patients. DSA reductions correlated histologically with reduced inflammation in transplant biopsies. Interestingly, ACR present in these patients also showed signs of resolution, hinting at direct effects on T-cell function in vivo. Similarly, Frohlich et al. reported resolution of symptoms and reduction in antibodies against double-stranded DNA (anti-dsDNA) following a single cycle of bortezomib therapy in a patient who developed multiple myeloma six years after diagnosis of systemic lupus erythematosus (SLE) [11]. The application of bortezomib in this unique patient was inspired based on an elegant study of bortezomib in mouse models of SLE, where inhibitory effects on the survival of short- and long-lived plasma cells were noted [12]. This preclinical work in SLE was led by Reinhard Voll, who, along with colleagues at multiple institutions, published clinical findings in 16 patients with treatment-refractory SLE who treated with bortezomib for 1–3 cycles of therapy [13]. Bortezomib treatment was associated with a rapid resolution of lupus symptoms including rash, arthritis, myositis, and serologic markers such as anti-dsDNA. The therapeutic effects attributed to bortezomib were evident up to six months after dosing cessation, suggesting a prolonged immunomodulatory effect. A significant decrease in plasma cells was found in most patients consistent with both preclinical data as well as a proposed mechanism of action based on findings in multiple myeloma [14]. Similar findings in patients with SLE have been reported by others where improvements in multiple organ manifestations of the disease were seen, including myocarditis and nephritis [15,16,17]. Of note, de Groot et al. monitored proteasome inhibition in a lupus patient treated with bortezomib and found high levels of inhibition of multiple immunoproteasome subunits [15]. The use of bortezomib has expanded to multiple B-cell driven autoimmune disorders, including IgA nephropathy, autoimmune hemolytic anemia, and neuromyelitis optica [18,19,20]. From these case studies, two major themes emerge. Bortezomib results in rapid resolution in disease symptoms in parallel to reductions in biomarkers of dysregulated plasma cell function (e.g., autoantibodies), however, treatment duration must be restricted due to safety concerns, namely the development of peripheral neuropathy [21]. Indeed, safety findings resulted in the early termination of the first and only randomized study of bortezomib in patients with SLE [22].

## 3. Therapeutic Potential of Selective Immunoproteasome Inhibitors

The collective clinical and preclinical experience of bortezomib as an anti-inflammatory agent created the basis for the discovery and development of novel proteasome inhibitors as immunomodulatory agents. The three approved proteasome inhibitors have as their primary targets the β5 subunit of the constitutive proteasome and the LMP7 subunit of the immunoproteasome, referred to as the chymotrypsin-like (CT-L) subunits based on their similar substrate preferences [23]. Cells express both forms of the proteasome with most tissues (e.g., heart) expressing mainly the constitutive proteasome while hematopoietic cells expressing mainly the immunoproteasome. However, plasma cells, both normal and transformed, express measurable levels of both proteasome forms, suggesting that either or both of the anti-myeloma and anti-inflammatory activity of bortezomib is driven by dual proteasome inhibition [24]. Exposure of myeloma cells to inhibitors selective for β5 and LMP7, either alone or in combination, revealed that induction of caspase 3 and 7 and thus apoptosis required dual inhibition of both CT-L subunits [24]. Selective inhibitors of LMP7 were not cytotoxic to myeloma, lymphoma, or non-transformed peripheral blood mononuclear cells (PBMC). These data initially dissuaded the development of selective inhibitors of LMP7 in oncologic disorders but did suggest that specific targeting of the immunoproteasome may provide a better safety profile in patients than dual proteasome inhibition. However, research involving preclinical models of hematologic malignancies such as lymphocytic leukemias has suggested anti-tumor potential for selective immunoproteasome inhibitors [25,26]. Inspired by these data, selective inhibitors of the immunoproteasome have been discovered and posited as anti-myeloma agents with improved safety profiles relative to bortezomib [27]. The most advanced of these agents, the dipeptide boronic acid M358 (Merck KGaA), showed >300-fold selectivity for LMP7 versus β5 and compelling activity in preclinical models of multiple myeloma [28,29]. However, a Phase 1 study in myeloma patients was terminated and the results of the study have not yet been published. 

The discovery of PR-957 (later ONX 0914) enabled researchers to probe the role of the immunoproteasome in therapeutically relevant in vitro and in vivo models of autoimmunity [30]. ONX 0914, a tripeptide analog of the tetrapeptide epoxyketone carfilzomib, was discovered in a medicinal chemistry campaign using purified proteasomes and cell lines and monitored with both enzymatic and active site occupancy assays in order to thoroughly determine potency and selectivity. Depending upon the assay and experimental system, ONX 0914 demonstrated 15–40-fold selectivity for LMP7 vs. β5 and roughly equivalent potency for binding to LMP2 as β5 [30,31]. Discovery efforts leading to ONX 0914 focused on tripeptide analogs of carfilzomib, based in part on parallel work designed to generate orally bioavailable peptide epoxyketone-based dual proteasome inhibitors for use in multiple myeloma [32]. Here, researchers found that tripeptide epoxyketones showed a minimal loss in potency relative to carfilzomib but enabled flexibility in subunit targeting. In particular, compounds containing a leucine at position one (P1) demonstrated increased potency for β5 vs. LMP7. ONX 0914 and other LMP7 selective analogs such as PR-924 contain a bulkier tyrosine residue at P1. Later crystallographic work demonstrated the selectivity of ONX 0914 for LMP7 over β5 was driven in large part by the interactions of the P1 tyrosine residue in ONX 0914 and the S1 pocket of LMP7 [31]. The S1 pocket of β5 is smaller requiring greater energy to move a methionine at position 45 and thus creating a differential in binding kinetics reflected as reduced potency. 

Armed with a tool compound that could selectively target the immunoproteasome in vitro and in vivo and assays that could translate subunit inhibition to the biologic outcome, researchers have built a strong case that selective targeting of the immunoproteasome could have a significant benefit in the treatment of autoimmune and immune-mediated disorders. This body of evidence is reviewed in more detail elsewhere in this issue and includes a review of the therapeutic activity of ONX 0914 seen in models of inflammatory arthritis, inflammatory bowel disease (IBD), SLE and lupus nephritis (LN), experimental autoimmune encephalomyelitis (EAE), and myasthenia gravis. In addition, salutary effects of immunoproteasome inhibition were seen both solid organ and allogeneic bone marrow transplant studies as well as acute inflammatory settings such as those seen with viral infection [33,34]. Ex vivo and in vitro studies revealed that ONX 0914 impacted plasma cell function, primarily through effects on migration, adhesion, and survival factors rather than direct induction of cell death [35,36]. In addition, ONX 0914 blocked inflammatory T-cell subsets (i.e., Th1 and Th17), increased regulatory T-cell function, and inhibited the release of proinflammatory cytokines from monocytes and dendritic cells, suggesting a broad impact on immune cell function [37,38]. Of particular importance, these effects on the innate and adaptive immune response did not come as a result of frank immunosuppression, as viral clearance in mice treated with ONX 0914 occurred with normal kinetics, and no long-term effects were seen on peripheral lymphocyte subsets [30,39]. Aside from these key findings around the mechanism of action and activity in disease models, ONX 0914 demonstrated adequate overall safety and tolerability in animals as well as activity with infrequent subcutaneous dosing, indicating a potentially favorable clinical profile suitable for use as a chronic therapy [30]. From this body of work, the stage was set for the clinical development of selective inhibitors of the immunoproteasome. 

## 4. Discovery and Early Development of KZR-616

**Figure 2:**
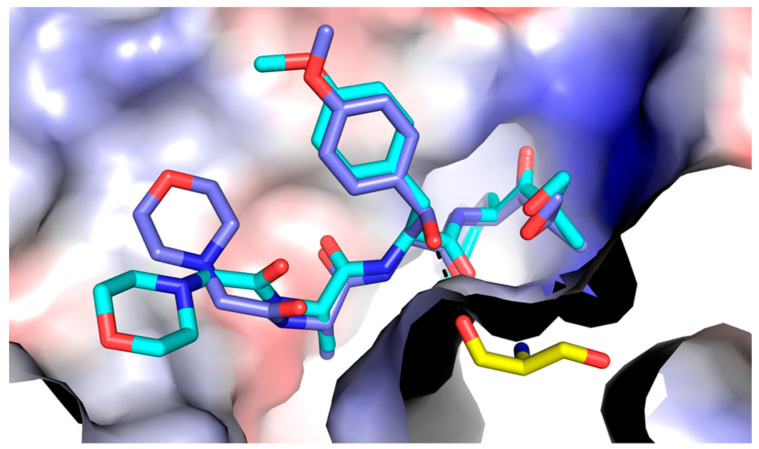
Binding modes of KZR-616 (purple) and ONX 0914 (blue) in the LMP7/β6 binding site of the human immunoproteasome. Adapted from [40].

Though highly useful as a tool compound, ONX 0914 demonstrated poor pharmaceutical properties, in particular low solubility, that precluded development in the clinical setting [40]. As a result, discrete discovery campaigns were undertaken with the goal of obtaining highly selective inhibitors of the immunoproteasome with sufficient additional properties that would enable clinical development as a chronically administered agent. Researchers at Onyx Pharmaceuticals and later Kezar Life Sciences focused on tripeptide epoxyketone analogs of ONX 0914, while those at Principia Biopharma utilized novel reversible covalent chemistry to target an active site cysteine residue found exclusively in LMP7 [41]. Both campaigns generated highly selective inhibitors of LMP7, KZR-329, and PRN1126, that demonstrated equivalent potency for this subunit but improved selectivity versus the other immunoproteasome subunits relative to ONX 0914. Surprisingly, these selective inhibitors of LMP7 had a muted anti-cytokine profile in vitro relative to ONX 0914 and were ineffective in mouse models of inflammatory arthritis, IBD, and EAE. However, when combined with selective inhibitors of LMP2 such as KZR-504 or LU-001 [42], the anti-inflammatory activity in vivo was equivalent to that of ONX 0914 [40,41]. These data suggested that inhibition of multiple immunoproteasome subunits is required for an optimal anti-inflammatory response. As a result of these findings, researchers at Kezar Life Sciences focused on developing LMP7/LMP2 inhibitors based on the peptide epoxyketone chemistry of ONX 0914, ultimately resulting in KZR-616 (Figure 2). 

In addition to the preferred subunit inhibition profile of immunoproteasome active sites, KZR-616 was also optimized for pharmacokinetic and metabolic properties in order to facilitate clinical development as a potential chronic therapy. Utilization of the ketoepoxide pharmacophore was employed to reduce the risk of off-target activity. Using global proteomic approaches with both active site and chemical probes, carfilzomib and other peptide epoxyketones have been shown to have extremely low reactivity against other classes of hydrolases and a low overall chemical reactivity [43,44]. Secondly, pharmacokinetic properties were taken into account in the design of KZR-616 to reduce potential toxicity risks of prolonged drug exposure. In animal models, KZR-616 and related analogs demonstrated rapid clearance from the plasma, similar to that reported for carfilzomib [45]. Given the risk of off-target activity for covalent inhibitors, reduced exposure time is considered a desirable physicochemical property, even for those with minimal off-target reactivity [46,47]. Finally, learnings from the metabolic profile of carfilzomib were applied during the discovery of KZR-616. In humans, peptide hydrolysis was one of the means of metabolism of carfilzomib, and though these peptides were of low toxicologic risk, clearance was highly variable in patients, especially in the setting of renal impairment [48,49]. KZR-616 was found to be resistant to peptide hydrolysis in preclinical studies with only a single, inactive metabolite present [45]. This metabolite, called the diol, was formed in a non-saturable fashion solely via microsomal epoxide hydrolase (mEH), a ubiquitously expressed enzyme that also mediates epoxide hydrolysis of carfilzomib [50]. Though analogs of KZR-616 were discovered that had reduced susceptibility to mEH, KZR-616 provided an optimal balance of metabolic stability and target inhibition profile [45]. Therefore, KZR-616 represents a drug candidate with exquisite selectivity for target subunits within the immunoproteasome and a pharmacologic profile that minimizes variations in exposure and potential drug-drug interactions.

**Figure 3:**
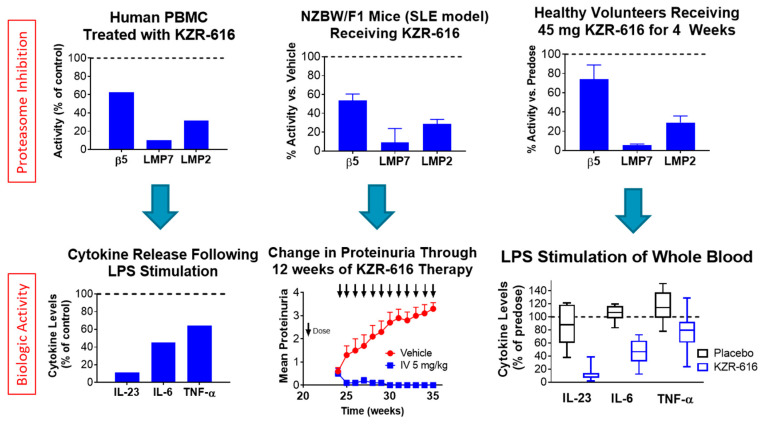
Comparison of Proteasome Subunit Inhibition and Biologic Activity in Preclinical Models to Clinical Pharmacodynamics and Biomarker Activity. Upper panels represent inhibition of selected proteasome subunits following in vitro exposure or single-dose administration to mice or healthy volunteers. Bottom panels represent readouts of cytokine release from endotoxin stimulation of human PBMC in vitro, proteinuria levels in NZB/W F1 mice, and ex vivo stimulation of whole blood and cytokine measurements in healthy volunteers. Data adapted from [40,51,55].

KZR-616 entered clinical trials in 2016 with a study of single and repeat dosing in healthy volunteers [51]. This study represented not only the first-in-man study of a selective inhibitor of the immunoproteasome but the first study of any proteasome inhibitor in healthy individuals. Subcutaneous administration of KZR-616 was found to be safe and tolerated in healthy men and women, demonstrated low inter-subject variability in drug exposure, and was ~100% bioavailable. The pharmacokinetic profile was one of rapid absorption and clearance with only the diol metabolite identified, as predicted from preclinical studies. KZR-616 resulted in selective inhibition of the immunoproteasome and target inhibition levels which matched or exceeded those seen with bortezomib or carfilzomib in myeloma patients [43,52]. In subjects receiving four weekly administrations of KZR-616 at either 30 or 45 mg, no serious adverse events (SAEs) were reported and no laboratory abnormalities were observed. This is in contrast to the frequent hematologic changes such as anemia, neutropenia, and thrombocytopenia seen with dual proteasome inhibitors in myeloma patients [53]. The most common reported adverse events of KZR-616 were related to effects at the injection site including erythema and induration and were mild to moderate in severity. Similar injection site reactions have been reported for bortezomib and thus may be related to localized high levels of proteasome inhibition [54]. In addition, no indication of cardiovascular toxicities, changes to renal or hepatic function, or signs of peripheral neuropathy were noted. The immunomodulatory effect of KZR-616 was assessed in these healthy subjects via ex vivo stimulation of whole blood samples with endotoxin and cytokine analysis. Following the fourth weekly dose of either KZR-616 or placebo, ex vivo endotoxin stimulation assays demonstrated reduced cytokine production in KZR-616 but not placebo-treated subjects indicating specific modulation of immune function. Indeed, the profile of subunit inhibition and immune effector cell changes seen in this healthy volunteer study matched that seen in preclinical studies (Figure 3). 

**Figure 4:**
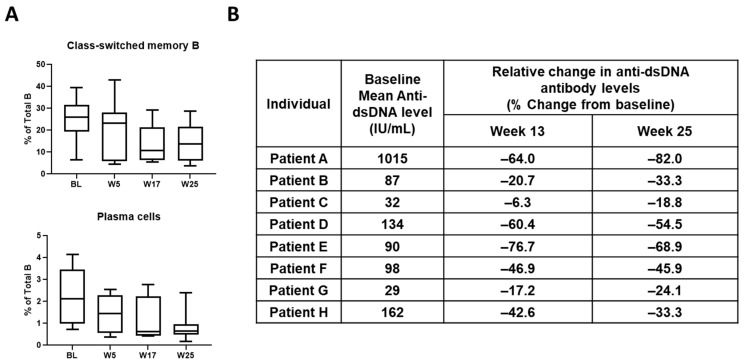
Biomarker Changes in Patients with SLE Treated with KZR-616. A. Flow cytometric analysis of class-switched memory B-cells (CD3^−^CD19^−^IgD^−^CD27^+^) and plasma cells (CD3^−^CD19^+^CD20^−^CD27^hi^) from start of treatment (BL) through Week (W) 25. B. Changes in plasma levels of autoantibodies relative to baseline at Weeks 13 (end of treatment) and 25 (end of study). Adapted from [56,57].

The safety and clinical activity of KZR-616 is currently being evaluated in Phase 2 studies in patients with lupus nephritis (LN), polymyositis (PM), and dermatomyositis (DM). In the MISSION study (NCT03393013), weekly administration of KZR-616 is being tested in patients with active proliferative LN. This trial also contained an open-label, dose escalation Phase 1b portion in SLE patients with or without nephritis. The safety profile reported in 47 patients treated with weekly doses of KZR-616 at 45, 60, and 75 mg administered for up to 13 weeks with a 12-week follow-up period was similar to that reported in healthy volunteers [56]. No clinically significant laboratory abnormalities were observed, and tolerability was similar at all tested dose levels. Patients enrolled in this study were taking stable background medications, including immunosuppressive agents (e.g., methotrexate) and corticosteroids with no signs of drug–drug interactions and a relatively low rate of infections during the six months of the study (<25% overall infection rate). In addition to these encouraging safety findings, all patients who received 13 weeks of treatment with KZR-616 showed at least some improvement across diseases measures, such as the Systemic Lupus Erythematosus Disease Activity Index 2000 (SLEDAI-2K), despite enrolling in the study with active disease. Reductions in circulating class switched B-cells and plasma cells were observed and in all eight patients with an abnormal level of anti-dsDNA antibodies at baseline, reductions at either Week 13 (end of treatment) and/or Week 25 (end of study) were observed (Figure 4) [56,57]. Finally, in two of two patients with active, proliferative LN enrolled in the Ph 1b portion of the study, improvements in proteinuria were seen within 17 weeks from treatment initiation. These rapid responses to both biomarkers of disease and clinically meaningful organ improvement served as motivation for the initiation of the Phase 2 portion of the study focused on patients with active, treatment-refractory LN. In the PRESIDIO study (NCT04033926), weekly administration of KZR-616 for 16 weeks at 45 mg is being tested in patients with either PM or DM. This study was preceded by a preclinical assessment of the therapeutic potential of immunoproteasome inhibitors in the C-protein Induced Myositis (CIM) model. Both ONX 0914 and KZR-616 induced an improvement in muscle function in mice when dosing was initiated two weeks after mice were immunized against muscle C protein [58]. Topline results from both of these clinical trials are expected in 2022. 

## 5. Discussion

Via impact on both the innate and adaptive immune response, selective inhibition of the immunoproteasome represents a novel and potentially broad approach to treating autoimmune diseases and immune-mediated disorders. In addition, the rapid immunomodulatory response to agents such as KZR-616 seen in patients along with a favorable safety and tolerability profile, suggest the use of such inhibitors in both the acute and chronic setting. These agents represent potentially significant improvements in both safety and/or efficacy relative to standard immunosuppressive therapies or other experimental therapeutics that target pathways with more limited molecular sequelae such as inhibitors of Bruton’s Tyrosine Kinase (BTK). KZR-616 represents the first selective inhibitor of the immunoproteasome to reach clinical testing in autoimmune diseases and early clinical results have faithfully replicated those seen in preclinical studies. Continued successful clinical trial results of KZR-616 will foster the expansion of the application of this agent to other immune-mediated disorders and may also foster the development of immunoproteasome inhibitors such as M358 in autoimmune diseases. Indeed, a drug discovery and development program based on reversible peptidomimetics that show selective inhibition of the immunoproteasome in vitro and therapeutic activity in mouse models of cardiac transplant has recently been initiated [59,60]. The wealth of chemistries available to selectively target the immunoproteasome and knowledge of proteasome biology built on decades of extensive research and clinical trials gives optimism that this new class of therapeutics will have as big an impact in the treatment of immune-mediated disorders as the dual proteasome inhibitors have had in B-cell neoplasms. Given that there are >80 autoimmune diseases and up to 23.5 million people living with an immune-mediated disorder in the United States alone, the impact from the successful development of these agents has far-reaching potential.
